# Scoring cytokine storm by the levels of MCP-3 and IL-8 accurately distinguished COVID-19 patients with high mortality

**DOI:** 10.1038/s41392-020-00433-y

**Published:** 2020-12-14

**Authors:** Liting Chen, Gaoxiang Wang, Jiaqi Tan, Yang Cao, Xiaolu Long, Hui Luo, Qing Tang, Tiebin Jiang, Wei Wang, Jianfeng Zhou

**Affiliations:** 1 Department of Hematology, Tongji Hospital, Tongji Medical College, Huazhong University of Science and Technology, No. 1095 Jiefang Avenue, 430030 Wuhan, Hubei China; 2 Department of Pediatrics, Tongji Hospital, Tongji Medical College, Huazhong University of Science and Technology, No. 1095 Jiefang Avenue, 430030 Wuhan, Hubei China; 3 Department of Laboratory Medicine, Tongji Hospital, Tongji Medical College, Huazhong University of Science and Technology, No. 1095 Jiefang Avenue, 430030 Wuhan, China; 4 Hematology Department of The Third Xiangya Hospital of Central South University, 410013 Changsha, China; 5 Department of Neurology, Tongji Hospital, Tongji Medical College, Huazhong University of Science and Technology, No. 1095 Jiefang Avenue, 430030 Wuhan, Hubei China

**Keywords:** Immunology, Biomarkers

**Dear Editor**,

Coronavirus disease 2019 (COVID-19) is a global-spread infectious disease caused by a novel coronavirus, SARS-CoV-2.^1^ COVID-19 causes heterogeneous disease phenotype, of which most patients exhibit mild to moderate symptoms, and ~15% progress to severe pneumonia, 5% of whom were eventually admitted to the intensive care unit (ICU) due to acute respiratory distress syndrome (ARDS), septic shock and/or multiple organ failure, and exhibit a high mortality rate. Two major obstacles to the improvement of the clinical outcomes in these patients are inadequate identification of the determinant cytokines correlated with fatal outcomes, and an inability to determine whether an individual patient has high risks of ICU admission and fatality.

Cytokine storm is featured in severe SARS-CoV-2 infection, and is proposed to be associated with an inferior clinical prognosis and lethal multiple organ dysfunction syndrome. Recent studies had identified MCP-3, IP-10, IL-6, and other cytokines as good predictors for the progression of COVID-19, while no algorism to computer the probability of ICU admission was available.^2,3^ Identification of the important cytokines and development of a uniform evaluation standard for cytokine storm are important to optimize clinical management.

A total of 242 ICU/non-ICU patients with COVID-19 were assigned into training (*n* = 136), internal test (*n* = 45), and external test (*n* = 61) sets. While the median time from illness onset to enrollment among all three sets was similar (Supplementary Table S1).

Serum samples were prospectively collected within the first 72 h of hospitalization. We initially conducted a multiplex screen for 48 cytokines in a total of 181 COVID-19 patients (training and internal test sets). The cytokine profile was dramatically different between ICU and non-ICU patients. The levels of 17 pro-inflammatory cytokines, including IL-8, IP-10, MCP-1, MCP-3, MIP-1α, IL-1α, IL-1ra, IL-6, IL-10, IL-16, IL-17A, IL-18, IFN-γ, M-CSF, β-NGF, HGF, and SCF were significantly increased in ICU patients. Meanwhile, the levels of 3 cytokines RANTES, GM-CSF, and TRAIL were significantly decreased in ICU patients compared to non-ICU patients (Supplementary Table S2).

The cytokine profiles in the ICU patients were further compared with those in patients with active bacterial septicemia (unpublished data generated by measuring electroluminescence) and patients with severe (grade 3–4) CRS due to anti-BCMA CAR-T cell therapy (Fig. 1a). In general, the cytokine responses to bacterial septicemia and CAR-T cells were much broader than those to SARS-CoV-2 infection. While profoundly elevated levels of IL-6 were consistently observed in COVID-19 ICU patients, bacterial sepsis, and CAR-T cell-induced CRS, increased levels of MCP-3 were found only in patients with COVID-19 or CAR-T cell-induced CRS but not in those with bacterial sepsis.

**Fig. 1:**
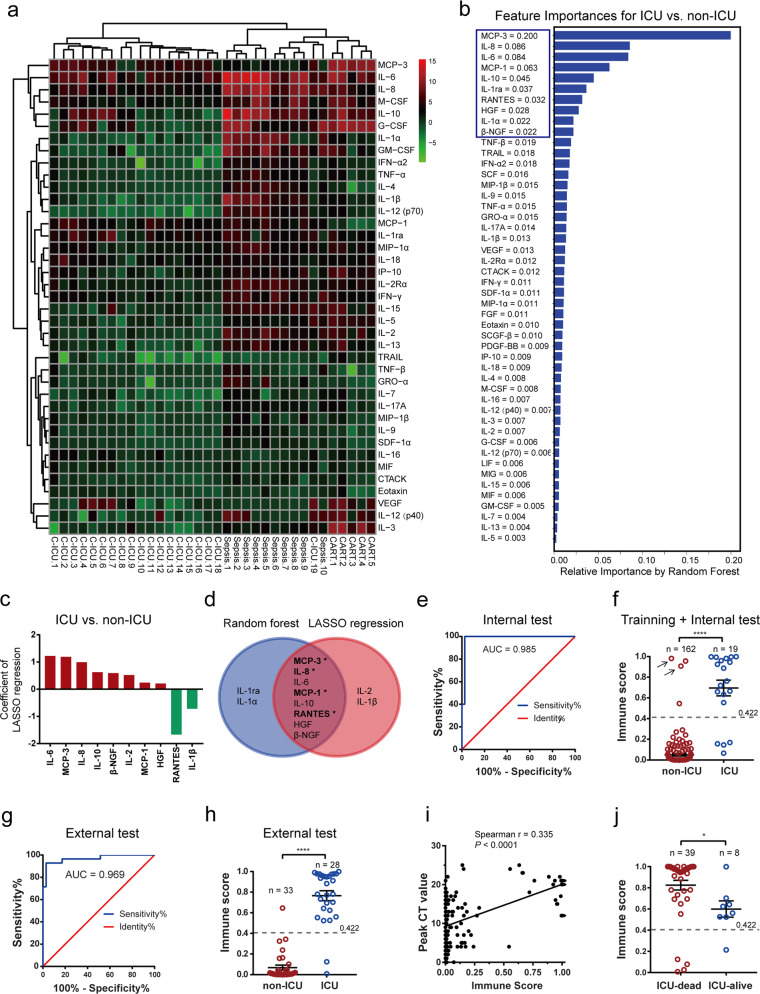
MCP-3/IL-8 scores were correlated with ICU admission and fatal outcomes. **a** Unsupervised clustering analysis of average baseline-adjusted and log2-transformed fold changes in cytokines in COVID-19 infected patients needing ICU admission, sepsis patients, and patients experiencing CRS after receiving CAR-T cell therapy. The 39 cytokines/chemokines presented overlap in all three sets of data. C-ICU, COVID-19 patients in ICU wards; sepsis, bacterial septicemia patients. **b** The most significant predictors for ICU admission were identified by random forest modeling. Relative variable importance is illustrated. **c** LASSO regression identified 10 analytes that contributed to distinguishing ICU patients from non-ICU patients. The coefficients are shown. Red bars and green bars represent cytokines positively and negatively contributing to the lasso regression model, respectively. **d** Venn diagram results for the random forest and lasso modeling are shown. *chemokines. **e** Receiver operating characteristic (ROC) curve evaluation of the performance of immune scores in identifying COVID-19 patients needing ICU admission in the internal test set. **f** Comparison of immune scores between the non-ICU and ICU patients in training and internal test sets. The arrows indicate patients who were transferred to ICU wards during the follow-up period. r, Spearman’s correlation coefficient. **g** ROC curve evaluation of the performance of immune scores in identifying COVID-19 patients needing ICU admission in the external test set. **h** Comparison of immune scores between the non-ICU and ICU patients in the external test sets. **i** Spearman’s correlation analyses of the MCP-3/IL-8 immune score and peak CT value. **j** Different outcomes (dead and alive) of ICU patients with significant differences in MCP-3/IL-8 immune scores. **P < 0.01; ****P < 0.0001. A *U* test was used

By utilizing the random forest model, we ranked the importance of 48 cytokines based on their ability to discriminate between ICU and non-ICU patients. MCP-3 ranked as the most important cytokine and was followed by IL-8, IL-6, MCP-1, IL-10, IL-1ra, RANTES, HGF, IL-1α, and β-NGF (Fig. 1b). Furthermore, bootstrap ranking LASSO regression identified a 10-cytokine set including IL-6, MCP-3, IL-8, IL-10, β-NGF, IL-2, MCP-1, HGF, RANTES, and IL-1β for discriminating between ICU and non-ICU patients (Fig. 1c).

A union set of the top 10 cytokines selected by the random forest model and LASSO regression algorithm including MCP-3, IL-8, IL-6, MCP-1, IL-10, RANTES, HGF, β-NGF, IL-1ra, IL-1α, IL-2, and IL-1β were entered in a logistic regression model with ICU/non-ICU admission as the dependent variable (Fig. 1d). We then used a forward stepwise variable selection approach to select the final predictors. Our final model identified the combination of MCP-3 and IL-8, used in combination, was the strongest predictor of ICU admission, with a *P*-value < 0.05. Furthermore, the coefficients calculated by the logistic regression model enabled the assignment of a weight to each cytokine and then a predicted probability of needing ICU admission for each individual patient could be obtained by summing up these weighted concentrations. The MCP-3/IL-8 algorithm was then employed to compute the probability of ICU admission by a mathematical equation: ln [p/(1−p)] = −9.0002 + 1.1985 × ihs (MCP-3) + 1.5457 × ihs (IL-8).

The performance of the MCP-3/IL-8 algorithm was evaluated by ROC analysis, the prediction cut-off was set at 0.422 in the internal test set by maximizing Youden’s index (Fig. 1e). The areas under the ROC curves (AUC) in the internal and external test set were 0.985 and 0.969, respectively (Fig. 1e, g). The positive predictive value and negative predictive value were calculated to be 83.3% (5/6) and 100% (39/39), respectively, in the internal test set. Moreover, four non-ICU patients at enrollment were identified to have high MCP-3/IL-8 scores, and two of them were eventually transferred to the ICU and died due to disease deterioration (Fig. 1f). The immune scores of ICU patients were significantly higher than those of non-ICU patients (Fig. 1f, h).

The difference in MCP-3/IL-8 scores was unlikely brought to be attributed to by the disparity in enrollment time (Supplementary Fig. S2a). The cytokine storm score of each individual patient was significantly correlated with peak CT score, oxygen demand, peak level of D-Dimer, and IFN-α2/IFN-γ (Fig. 1i and Supplementary Fig. S2b–d) while was not correlated with viral clearance time or peak anti-SARS-CoV-2 IgG levels (Supplementary Fig. S2e–f). Importantly, the patients who died in the ICU had significantly higher MCP-3/IL-8 scores than the surviving ICU patients (Fig. 1j).

The subgroup with high MCP-3/IL-8 score (range from 0.524 to 0.999) displayed a significantly higher probability of ICU admission and mortality compared with the subgroup with low MCP-3/IL-8 score (range from 0.000 to 0.343) in a total of 242 enrolled patients. Older age, male gender, and a lower ratio of IFN-α2/IFN-γ were found to be significantly associated with the high-scoring subgroup (Supplementary Table S3). On the other hand, illness enrollment time and comorbidities did not significantly differ between the two subgroups. Much more frequent use of treatments of corticosteroid (91.3 versus 44.9%, *P* < 0.0001), ECMO (10.9 versus 0.5%, *P* < 0.0001) and renal replacement therapy (43.5 versus 1%, *P* < 0.0001) in high-scoring subgroup might mirror higher morbidity rate of ARDS or multiple organ failure in this subgroup, and also suggesting these treatments might not interfere the computation of MCP-3/IL-8 algorithm. Furthermore, the degree of organic impairment, as evaluated by the MCP classification, NT-proBNP level, cTn I level, AKI stage, and qSOFA were significant higher in high-scoring patients (Table S3).

In biopsy and autopsy studies, pulmonary pathology for COVID-19 patients showed diffuse alveolar damage with mononuclear cells, and macrophages infiltrating air spaces.^4^ As the MCP proteins and IL-8 chemoattract and activate monocytes, activated T cells, neutrophil, and other immune cells,^5^ it is therefore reliable that finding overproduction of MCP proteins and IL-8 is linked to a lethal cytokine storm and pathological phenotypes.

In conclusion, this study identified chemokines and IL-6 as determinant cytokines in severe cytokine storm. The MCP-3/IL-8 score offered potentially clinically meaningful risk strata for clinical management by providing a uniform standard to evaluate the severity of cytokine storm in the individual patient setting, and distinguishing patients with fatal clinical outcomes. These findings advance previous work and help to optimize the current clinical management of COVID-19.

## Supplementary information

Supplemental material

## References

[1] Lu R (2020). Genomic characterisation and epidemiology of 2019 novel coronavirus: implications for virus origins and receptor binding. Lancet.

[2] Yang Y (2020). Plasma IP-10 and MCP-3 levels are highly associated with disease severity and predict the progression of COVID-19. J. Allergy Clin. Immunol..

[3] McElvaney OJ (2020). Characterization of the Inflammatory Response to Severe COVID-19 Illness. Am. J. Respir. Crit. Care Med..

[4] Li H (2020). SARS-CoV-2 and viral sepsis: observations and hypotheses. Lancet.

[5] Xu LL (1995). Monocyte chemotactic protein-3 (MCP3) interacts with multiple leukocyte receptors: binding and signaling of MCP3 through shared as well as unique receptors on monocytes and neutrophils. Eur. J. Immunol..

