# Supplementary material for: Scoring cytokine storm by the levels of MCP-3 and IL-8 accurately distinguished COVID-19 patients with high mortality
Source: Signal Transduct Target Ther. 2020 Dec 14;5:292. doi: 10.1038/s41392-020-00433-y (PMC7734384; doi:10.1038/s41392-020-00433-y)
Supplement: Supplementary file 1 — Supplemental material [file 41392_2020_433_MOESM1_ESM.docx]

Supplementary Materials for

Scoring Cytokine Storm by the Levels of MCP-3 and IL-8 Accurately Distinguished COVID-19 Patients with High Mortality

Liting Chen, Gaoxiang Wang, Jiaqi Tan, Yang Cao, Xiaolu Long, Hui Luo, Qing Tang, Tiebin Jiang, Wei Wang, Jianfeng Zhou.

Correspondence to: jfzhou@tjh.tjmu.edu.cn

**This PDF file includes:**

Materials and Methods

Figures. S1 to S2

Tables S1 to S3

Materials and Methods

Clinical specimens and study design

This study population including the training, internal test and external test set consisted of 242 participants with newly diagnosed COVID-19 between February 8, 2020, and February 29, 2020 in Tongji Hospital. Written informed consent was obtained from all patients, and waived if the patient was too unwell to provide it under an emerging infectious disease provision. This study was approved by the Medical Ethics Committee of Tongji Hospital, Tongji Medical College, Huazhong University of Science and Technology, Wuhan, China. One hundred eighty-one participants were from the wards managed by Tongji medical team, and randomly assigned (3:1) to the training set (n= 136) or internal test set (n= 45) using a random number generator. Sixty-one participants of an external test set were from medical wards independently managed by national medical teams. Serum samples were prospectively collected within the first 72 h of hospitalization outside or inside ICU according to the study design (Figure S1). Exclusion criteria included the following: (1) Lack of clinical and chest radiographic data; (2) no written informed consent could be obtained if the patient was not too unwell to provide it; and (3) enrollment in remdesivir or other treatment clinical trials. Patients’ data, including demographics, medical history, oximetric assessment, swabs monitoring, radiography and clinical outcomes were recorded in an electronic data capture system (EDCS). Medical data from electronic health records were extracted using a standardized data collection form. Each medical ward used standardized guidance to minimize variability in the diagnosis and estimation of the severity of COVID-19 pneumonia26. This study also included cryopreserved serum samples collected from 5 patients with grade 3-4 cytokine release syndrome (CRS) due to anti-BCMA chimeric antigen receptor (CAR) T cell therapy, 22 healthy donors. The previous unpublished data of cytokine levels from 10 bacterial septicemia patients were also included in this study.

Sample collection

Blood samples were collected and immediately transferred to a 4℃ refrigerator and processed within 24 hours. Serum samples were collected using a serum separator tube (SST) and allowed to clot for 30 minutes at room temperature before centrifugation for 15 minutes at 1,000 × g. The serum was harvested and assayed immediately or aliquoted, and samples were stored at ≤ -80 ℃. Repeated freeze-thaw cycles were avoided.

Cytokine measurements

The levels of serum cytokines were determined by Bio-Plex Pro Human Cytokines 48-Plex Screening assay (Bio-Rad Life Sciences, Hercules, CA, USA) using a Luminex FlEXMAP 3D system (Luminex, Austin, TX, USA) according to the manufacturer’s protocols. The 48-Plex Screening panel: Basic FGF, CTACK, eotaxin, G-CSF, GM-CSF, GRO-α, HGF, ICAM-1, IFN-α2, IFN-γ, IL-1α, IL-1ra, IL-2, IL-2Rα, IL-3, IL-4, IL-5, IL-6, IL-7, IL-8, IL-9, IL-10, IL-12, IL-13, IL-15, IL-16, IL-17A, IL-18, IP-10, LIF, MCP-1, MCP-3, M-CSF, MIF, MIG, MIP-1α, MIP-1β, β-NGF, PDGF-BB, RANTES, SCF, SCGF-β, SDF-1α, TNF-α, TNF-β, TRAIL, VCAM-1, VEGF-A. Data were analyzed using Bio-Plex Manager 6.2 software (Bio-Rad Life Sciences, Hercules, CA, USA). Undetected values were inputted with a random value between 0 and the limit of detection (LOD) to avoid an artificial reduction in the standard deviation.

Scoring CT images

Non-contrast enhanced chest CT scans were performed weekly, and if necessary, additional scans were also permitted. A semi-quantitative scoring system was used to estimate lung involvement according to the protocol of a previous study. Each of the 5 pulmonary lobes was visually scored from 0 to 5 depending on the range of the pulmonary involvement (0: no involvement; 1: <5% involvement; 2: 6%-25% involvement; 3: 26%-49% involvement; 4: 50%-75% involvement; 5: >75% involvement). The total score was the sum of these 5 lobe scores and ranged from 0 to 25. For each patient, the CT manifestation with the highest score was defined as the peak CT score and used for further analysis. All CT images of the 181 patients in our cohort were reviewed and scored independently by 2 radiologists who were blinded to the clinical features, and final scores were determined by consensus.

Mean oxygen score

Data on patients’ changes in oxygen-support requirements were assessed daily from enrollment to discharge on a seven-category ordinal scale. Patients with scores below 3 were defined as having no need for oxygen. For each patient, the mean score in oxygen-support requirements (MOSR) was calculated by the formula:

Viral clearance time and peak D-D dimer level

The virus clearance time was defined as the time from enrollment to the first day of two consecutive negative RT-PCR assays performed with nasopharyngeal swabs collected at least 24 h apart. The D-D dimer level in each patient was assessed weekly from enrollment to discharge. For each patient, the highest D-D dimer level was defined as the peak D-D dimer level and used for further analysis.

Determination of SARS-CoV-2-specific IgG

SARS-CoV-2-specific IgG was detected by a paramagnetic particle chemiluminescent immunoassay (CLIA) using an iFlash-SARS-CoV-2 IgG assay kit and iFlash Immunoassay Analyzer (Shenzhen YHLO Biotech Co., Ltd, China).

Statistical analysis

Two feature selection techniques, Least Absolute Shrinkage and Selection Operator (LASSO) and random forests were simultaneously employed to identify the most important cytokines in distinguishing ICU patients from non-ICU patients among correlated potential predictors from the training set alone. Inverse hyperbolic sine transformation was employed before statistical modeling. To avoid inconsistencies in terms of variable selection in the LASSO modeling process, a bootstrap ranking procedure was used, in which 1,000 bootstrap samplings were carried out, a LASSO estimates matrix representing variable ranking according to importance was generated, and an external intersection operation was conducted to extract an optimal set of predictors and obtain the robust selected predictors. The penalty parameter λ for LASSO was chosen using 10-fold cross-validation in this process. In the random forest modeling process, the number of decision trees in the forest was set at 2,500 and the importance of each cytokine was calculated using the decrease in node impurity weighted by the probability of reaching that node. The node probability was calculated as the number of samples that reached the node, divided by the total number of samples. The feature importances were then normalized to a value between 0 and 1 by dividing by the sum of all feature importance values, and the higher the value, the more important the cytokine was. Finally, a union set of cytokines was formulated based on the top 10 cytokines selected by either the LASSO or random forest method. These selected cytokines were entered into a logistic regression model with ICU/non-ICU as the dependent variable and a final model was developed using stepwise method to identify the cytokines significantly associated with ICU admission in the training set. In the resulting algorithm, every cytokine was assigned a weight computed by the model that best fit the training data. The sum of these weighted concentrations led to a predicted probability for each individual patient.

We calculated the misclassification error and the area under curve (AUC) of the receiver operating characteristic curve (ROC) for the internal test data as measures of the predictive performance of the fitted models. Youden index was calculated according to the ROC to help to define the appropriate cut-off value. All the analyses were performed using the statistical R version 3.6.3 software (www.r-project.org) and Python 3.7. The Univariate analyses were assessed with χ2 test of association for categorical values and the U test for continuous values. Correlations between immune scores and clinical characteristics were estimated by Spearman correlation analysis.

Figure. S1.

Flowchart of study design.

**
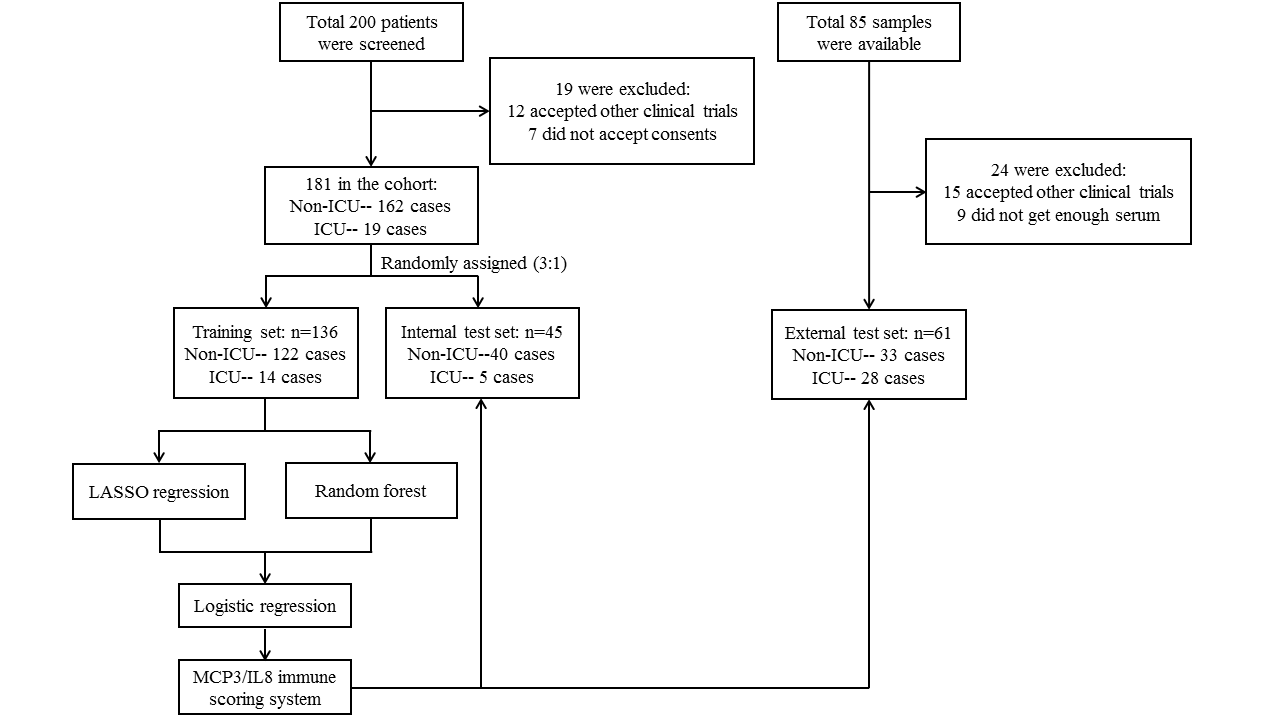
**

Figure. S2.

Spearman’s correlation analyses of the MCP-3/IL-8 immune score and clinical index.

a Illness onset time. b Mean oxygen score. c Peak D-Dimer level. d Ratio of IFN-α2/IFN-γ concentrations. e Viral clearance time. f Anti-SARS-CoV-2 IgG level. Type or paste caption here. Create a page break and paste in the Figure above the caption.

**
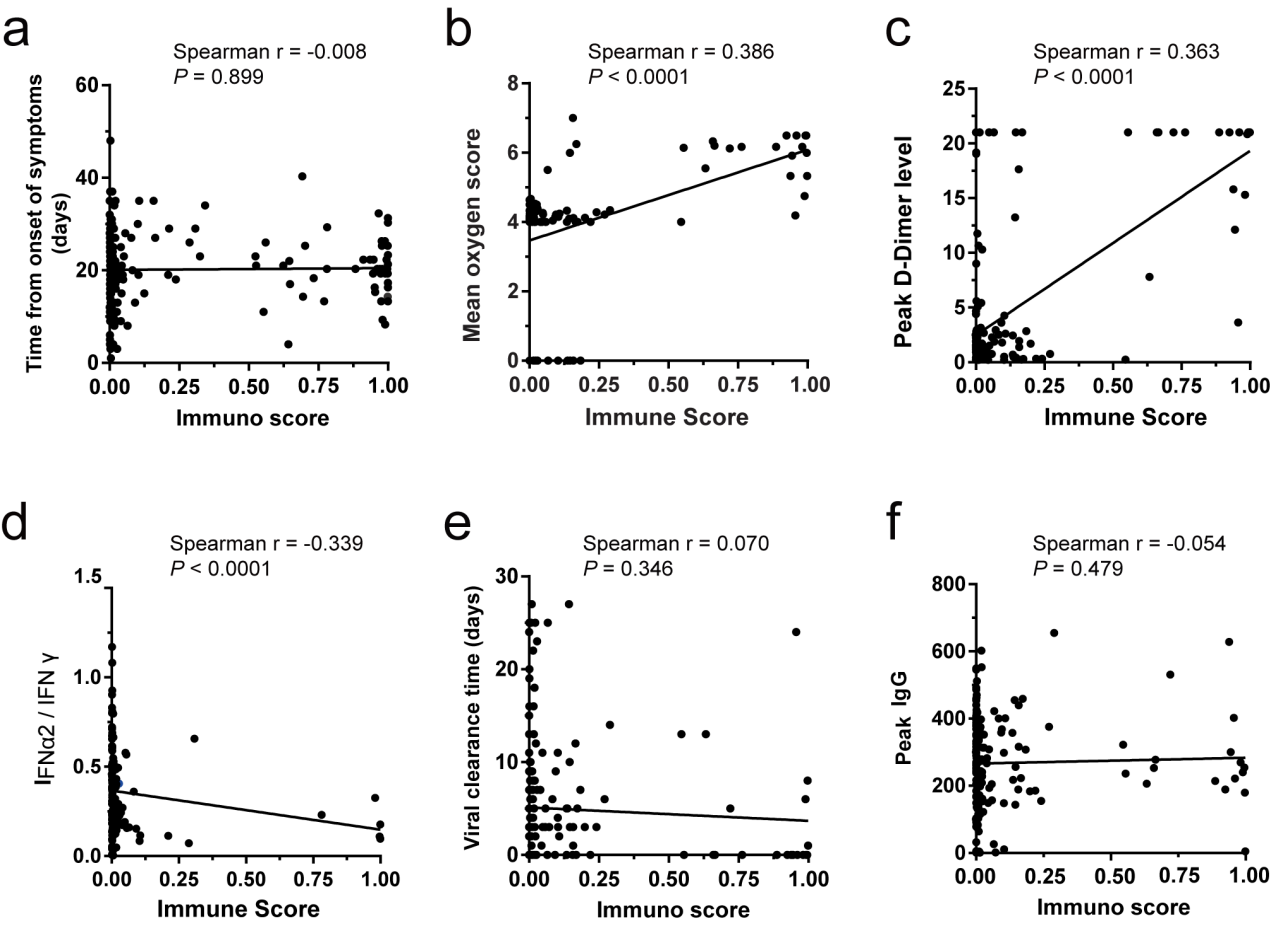
**

Table S1.

Demographic and Clinical Characteristics of the Patients

| Characteristics | Training Set (n=136) | Internal Test Set (n=45) | External test Set (n=61) | *P*-value^a^ | *P-*value^b^ |
| --- | --- | --- | --- | --- | --- |
| Age, median (IQR), y | 58 (39-68) | 62 (54-69) | 64 (55-70) | 0.0034 | 0.5718 |
| Sex |  |  |  |  |  |
| Male, No. (%) | 65 (47.8) | 21 (46.7) | 40 (65.6) | 0.0302 | 0.0730 |
| Comorbidity, No. (%) | 68 (50.0) | 26 (57.8) | 41 (67.2) | 0.0300 | 0.4153 |
| Hypertension | 34 (25.0) | 18 (40.0) | 23 (37.7) |  |  |
| Diabetes | 23 (16.9) | 10 (22.2) | 17 (27.9) |  |  |
| Coronary artery heart disease | 13 (9.6) | 3 (6.7) | 8 (13.1) |  |  |
| Cancer | 7 (5.1) | 2 (4.4) | 2 (3.3) |  |  |
| COPD | 3 (2.2) | 0 | 2 (3.3) |  |  |
| Time from illness onset to enrollment, median (IQR), d | 21 (13-25) | 19 (14-25) | 21 (16-26) | 0.4308 | 0.3126 |
| ICU care at enrollment, No. (%) | 14 (10.3) | 5 (11.1) | 28 (45.9) | < 0.0001 | 0.0010 |
| Treatment during study period, No. (%) |  |  |  |  |  |
| Antibiotics | 105 (77.2) | 34 (75.6) | 44 (72.1) | 0.4752 | 0.8243 |
| Antivirals^#^ | 119 (87.5) | 40 (88.9) | 54 (88.5) | 0.3032 | > 0.9999 |
| Corticosteroids | 67 (49.3) | 25 (55.6) | 38 (62.3) | 0.1221 | 0.5504 |
| Intravenous Immunoglobin | 28 (20.6) | 7 (15.6) | 20 (32.8) | 0.0742 | 0.0700 |
| ECMO | 2 (1.5) | 1 (2.2) | 3 (4.9) | 0.1734 | 0.6353 |
| Renal replacement therapy | 6 (4.4) | 3 (6.7) | 13 (21.3) | 0.0005 | 0.0535 |
| Highest level of respiratory support, No. (%) | 21 (15.4) | 7 (15.6) | 33 (54.1) | < 0.0001 | < 0.0001 |
| High-flow nasal cannula oxygen therapy | 1 (0.7) | 1 (2.2) | 4 (6.6) |  |  |
| Non-invasive mechanical ventilation | 5 (3.7) | 1 (2.2) | 4 (6.6) |  |  |
| Invasive mechanical ventilation | 15 (11.0) | 5 (11.1) | 25 (41.0) |  |  |
| Outcomes at data cutoff, No. (%) |  |  |  | < 0.0001 | 0.0050 |
| Discharged alive | 122 (89.7) | 39 (86.7) | 36 (59.0) |  |  |
| Died | 13 (9.6) | 6 (13.3) | 22 (36.1) |  |  |
| Hospitalization | 1 (0.7) | 0 | 3 (4.9) |  |  |
| Outcomes of non-ICU patients, No. | 122 | 40 | 33 | > 0.9999 | > 0.9999 |
| Discharged from hospital, No. (%) | 121 (99.2) | 39 (97.5) | 33 (100) |  |  |
| Transfer to ICU, No. (%) | 1 (0.8) | 1 (2.5) | 0 |  |  |
| Outcomes of ICU patient*, No. | 15 | 6 | 28 | > 0.9999 | > 0.9999 |
| Discharge alive, No. (%) | 1 (6.7) | 0 | 3 (10.7) |  |  |
| Died in ICU, No. (%) | 13 (86.7) | 6 (100) | 22 (78.6) |  |  |
| Still in ICU, No. (%) | 1 (6.7) | 0 | 3 (10.7) |  |  |
| Organic impairment |  |  |  |  |  |
| MCP classification median (IQR) | 5 (4-6) | 5 (5-7) | 7.5 (5-9) | < 0.0001 | 0.0038 |
| NT-proBNP level median (IQR) | 124 (38.75-360) | 177 (57.75-459.8) | 1146 (96-6312) | < 0.0001 | 0.0050 |
| cTn I level median (IQR) | 4.2 (2-9.6) | 5.05 (2.95-15.3) | 31.7 (3.4-241.9) | 0.0001 | 0.0533 |
| AKI stage median (IQR) | 0 (0-0) | 0 (0-0) | 0 (0-1) | 0.0001 | 0.0331 |
| qSOFA score median (IQR) | 1 (0-1) | 1 (0-1) | 2 (1-3) | < 0.0001 | < 0.0001 |

COPD denotes chronic obstructive pulmonary disease; ICU intensive care unit; ECMO Extracorporeal Membrane Oxygenation.

^a^ *P*-value for comparison of training set to external test set;

^b^ *P*-value for comparison of internal test to external test set;

^#^ Abidol hydrochloride and/or Oseltamivir;

^*^ Two of non-ICU patients at enrollment (1 in the training set and the other one in the internal test set) were transferred to ICU because of clinical deterioration, and eventually died in ICU.

Table S2.

Comparison of cytokines in different groups of patients

| **Analyte** | **Healthy (n=22)** | **Total COVID-19 (n=181)** | **Non-ICU (162)** | **ICU (n=19)** | **Total COVID-19 versus Healthy** | **ICU versus non-ICU** |
| --- | --- | --- | --- | --- | --- | --- |
|  | mean (s.e.m.) | mean (s.e.m.) | mean (s.e.m.) | mean (s.e.m.) | *P-* value^a,b^ | *P-* value^a,b^ |
| **Chemokine** |  |  |  |  |  |  |
| CTACK | 585.73 (37.70) | 1343.38 (101.43) | 1418.97 (110.45) | 698.94 (155.27) | 0.2976 | 0.207 |
| Eotaxin | 73.77 (4.87) | 140.78 (13.03) | 146.65 (14.43) | 90.76 (11.51) | 0.3830 | 0.767 |
| GRO-α | 828.68 (9.86) | 1510.58 (102.38) | 1595.60 (111.21) | 785.64 (150.34) | 0.4963 | 0.076 |
| IL-8 | 6.21 (0.84) | 29.43 (7.25) | 12.41 (1.50) | 174.55 (59.41) | 0.8476 | **<0.0001** |
| IP-10 | 472.99 (38.72) | 1629.05 (411.94) | 1594.21 (456.87) | 1926.10 (491.46) | 0.3872 | **0.008** |
| MCP-1 | 34.38 (2.24) | 168.46 (28.30) | 110.52 (19.48) | 662.48 (179.52) | **0.0241** | **<0.0001** |
| MCP-3 | 0.16 (0.01) | 1.67 (0.43) | 0.89 (0.40) | 8.29 (1.64) | 0.2380 | **<0.0001** |
| MIG | 152.48 (8.19) | 399.62 (53.32) | 386.85 (56.57) | 508.44 (161.36) | 0.4559 | 0.115 |
| MIP-1α | 2.61 (0.16) | 10.40 (0.83) | 9.81 (0.84) | 15.46 (3.22) | **0.0019** | **0.017** |
| MIP-1β | 264.54 (5.52) | 639.29 (43.48) | 672.59 (47.41) | 355.36 (60.66) | **0.0302** | 0.355 |
| RANTES | 16424.37 (1073.75) | 27175.39 (1940.48) | 29250.44 (2092.98) | 9482.93 (2281.74) | 0.9448 | **<0.0001** |
| SDF-1α | 845.28 (34.07) | 930.35 (55.57) | 948.30 (60.98) | 777.29 (95.51) | 0.1786 | 0.904 |
| **Interleukin** |  |  |  |  |  |  |
| IL-1α | 12.78 (1.53) | 8.17 (0.82) | 7.62 (0.86) | 12.91 (2.47) | **<0.0001** | **0.002** |
| IL-1β | 4.05 (0.26) | 7.02 (0.60) | 7.39 (0.66) | 3.92 (1.09) | 0.2641 | 0.400 |
| IL-1ra | 157.13 (12.58) | 1246.92 (244.67) | 769.30 (91.79) | 5319.26 (2007.74) | **0.0005** | **<0.0001** |
| IL-2 | 1.37 (0.18) | 2.16 (0.40) | 2.16 (0.44) | 2.20 (0.52) | 0.4432 | 0.077 |
| IL-2Rα | 60.14 (2.97) | 251.05 (29.41) | 254.65 (32.08) | 220.40 (62.17) | **0.0352** | 0.091 |
| IL-3 | 0.06 (0.01) | 0.21 (0.12) | 0.21 (0.14) | 0.15 (0.07) | 0.2060 | 0.760 |
| IL-4 | 7.07 (0.29) | 13.23 (0.91) | 13.88 (0.99) | 7.66 (1.37) | 0.6323 | 0.298 |
| IL-5 | 1.91 (0.30) | 26.01 (18.73) | 27.91 (20.91) | 9.72 (7.04) | **0.0057** | 0.650 |
| IL-6 | 1.37 (0.27) | 29.24 (10.17) | 7.85 (1.79) | 211.60 (86.84) | 0.2327 | **<0.0001** |
| IL-7 | 14.74 (2.24) | 14.19 (2.28) | 14.27 (2.52) | 13.51 (3.66) | **0.0006** | 0.073 |
| IL-9 | 391.13 (6.40) | 1006.04 (74.89) | 1071.21 (81.39) | 450.35 (99.48) | 0.3012 | 0.148 |
| IL-10 | 0.70 (0.07) | 5.26 (2.05) | 1.04 (0.12) | 41.25 (17.82) | 0.3573 | **0.002** |
| IL-12 (p70) | 3.18 (0.71) | 2.95 (1.52) | 3.14 (1.69) | 1.30 (0.37) | **0.0006** | 0.617 |
| IL-12 (p40) | 8.76 (0.79) | 71.19 (27.08) | 68.60 (29.58) | 93.27 (55.59) | 0.9479 | 0.904 |
| IL-13 | 1.40 (0.23) | 2.45 (0.34) | 2.54 (0.38) | 1.72 (0.30) | 0.7410 | 0.448 |
| IL-15 | 4.40 (0.77) | 66.35 (30.76) | 67.65 (34.15) | 55.29 (34.94) | **0.0087** | 0.767 |
| IL-16 | 61.07 (4.43) | 174.69 (15.04) | 170.44 (16.19) | 210.95 (38.68) | 0.1932 | **0.021** |
| IL-17A | 12.61 (1.29) | 12.15 (1.34) | 12.12 (1.49) | 12.40 (1.82) | **0.0050** | **0.032** |
| IL-18 | 38.00 (1.94) | 225.67 (33.56) | 196.03 (23.51) | 478.43 (247.35) | **0.0003** | **0.013** |
| LIF | 31.54 (4.84) | 92.52 (9.11) | 95.09 (9.93) | 70.63 (18.98) | **0.0263** | 0.972 |
| **Interferon** |  |  |  |  |  |  |
| IFN-α2 | 15.94 (0.81) | 21.33 (1.51) | 21.89 (1.63) | 16.51 (3.30) | 0.6907 | 0.614 |
| IFN-γ | 37.82 (3.58) | 111.79 (10.92) | 105.31 (11.61) | 167.06 (29.70) | 0.4747 | **0.002** |
| **CSF** |  |  |  |  |  |  |
| G-CSF | 5.08 (0.89) | 52.34 (11.39) | 42.93 (9.51) | 132.59 (71.16) | 0.6894 | 0.054 |
| GM-CSF | 0.80 (0.27) | 3.95 (1.52) | 4.23 (1.69) | 1.56 (0.65) | 0.4699 | **0.025** |
| M-CSF | 10.70 (0.78) | 63.48 (6.99) | 62.44 (7.61) | 72.36 (15.24) | **0.0028** | **0.018** |
| **TNF** |  |  |  |  |  |  |
| TNF-α | 75.01 (1.77) | 153.80 (10.66) | 161.01 (11.63) | 92.29 (16.18) | 0.6474 | 0.713 |
| TNF-β | 446.59 (8.63) | 1186.65 (86.03) | 1261.38 (93.55) | 549.47 (111.37) | **0.0390** | 0.141 |
| TRAIL | 27.53 (2.03) | 53.41 (5.00) | 57.26 (5.45) | 20.55 (7.10) | 0.5285 | **0.025** |
| **Others** |  |  |  |  |  |  |
| β-NGF | 0.28 (0.05) | 0.49 (0.09) | 0.42 (0.09) | 1.16 (0.31) | 0.8718 | **0.019** |
| Basic FGF | 70.40 (2.26) | 113.13 (7.84) | 118.50 (8.56) | 67.42 (12.26) | 0.1150 | 0.643 |
| HGF | 304.19 (22.42) | 1096.79 (131.01) | 900.02 (71.09) | 2774.53 (1036.86) | **0.0094** | **<0.0001** |
| MIF | 1429.16 (159.67) | 2685.93 (234.31) | 2721.27 (257.39) | 2384.59 (414.90) | 0.8597 | 0.140 |
| PDGF-BB | 2354.93 (209.05) | 5589.81 (543.15) | 5892.27 (594.50) | 3010.89 (858.70) | 0.7121 | 0.090 |
| SCF | 60.55 (2.52) | 183.29 (14.22) | 178.28 (14.98) | 226.00 (45.21) | **0.0072** | **0.032** |
| SCGF-β | 98368.04 (5508.67) | 362846.80 (28718.86) | 380159.52 (31600.72) | 215233.12 (32575.14) | **0.0002** | 0.813 |
| VEGF-A | 2.90 (0.35) | 81.23 (17.73) | 78.87 (19.20) | 101.31 (42.65) | 0.1329 | 0.667 |
| ^a^Bold text indicates *P*-values < 0.05. Red cells represent upregulated analytes and green cells represent downregulated analytes (two-group comparisons). ^b^ Mann-Whitney U test with two independent groups (healthy vs. total COVID-19, and non-ICU vs. ICU). | | | | | | |
|  |  |  |  |  |  |  |
|  |  |  |  |  |  |  |

Table S3.

| Comparison of cytokines in different groups of patients | | |  |  |
| --- | --- | --- | --- | --- |
| Characteristic | low scoring (n=196)  ≤ 0.422 | high scoring (n=46)  >0.422 | *P*-value |  |
| Age, years, median (range) | 59 (17-87) | 67 (42-86) | **<0.0001 ^a^** |  |
| Sex, No. (%) |  |  | **0.0208 ^b^** |  |
| Female | 101 (51.5) | 15 (32.6) |  |  |
| Male | 95 (48.5) | 31 (67.4) |  |  |
| Comorbidity, No. (%) |  |  |  |  |
| Hypertension | 58 (29.6) | 17 (37) | 0.331 ^b^ |  |
| Diabetes | 38 (19.4) | 12 (26.1) | 0.3125 ^b^ |  |
| Coronary artery heart disease | 17 (8.7) | 7 (15.2) | 0.1814 ^b^ |  |
| Cancer | 9 (4.6) | 2 (4.3) | 1.0000 ^b^ |  |
| COPD | 3 (1.5) | 2 (4.3) | 0.2415 ^b^ |  |
| Time from illness onset to enrollment, median (range) | 21 (1-48) | 20 (4-32) | 0.7616 ^a^ |  |
| Treatment, No. (%) |  |  |  |  |
| Antivirals | 172 (87.8) | 41 (89.1) | 0.7960 ^b^ |  |
| Corticosteroids | 88 (44.9) | 42 (91.3) | **<0.0001 ^b^** |  |
| ECMO | 1 (0.5) | 5 (10.9) | **<0.0001 ^b^** |  |
| Renal replacement therapy | 2 (1) | 20 (43.5) | **<0.0001 ^b^** |  |
| Death (%) | 5 (2.6) | 36 (78.3) | **<0.0001 ^b^** |  |
| IFN-α2/IFN-γ, median (range) | 0.33 (0-7.08) | 0.15 (0-0.42) | **<0.0001 ^a^** |  |
| Organic impairment |  |  |  |  |
| MCP classification median (range) | 5 (3-11) | 9 (4-12) | **<0.0001 ^a^** |  |
| NT-proBNP level median (range) | 109 (5-39875) | 7443 (9-70000) | **<0.0001 ^a^** |  |
| cTn I level median (range) | 3.75 (0.9-1821) | 241.9 (1.9-12553) | **<0.0001 ^a^** |  |
| AKI stage median (range) | 0 (0-3) | 1.5 (0-3) | **<0.0001 ^a^** |  |
| qSOFA score median (range) | 1 (0-3) | 3 (0-3) | **<0.0001 ^a^** |  |
| Data are median (range) or n (%); Bold text indicates *P*-values < 0.05, ^a^ Mann-Whitney U test is used for continuous variables and ^b^ chi-square test is used for classified variables; COPD denotes chronic obstructive pulmonary disease and ECMO Extracorporeal Membrane Oxygenation. | | | |  |
|  |  |  |  |  |
|  |  |  |  |  |
